# Obstructive or non-obstructive megacystis: a prenatal dilemma

**DOI:** 10.3389/fped.2024.1379267

**Published:** 2024-07-02

**Authors:** Martina Mandaletti, Elisa Cerchia, Elena Ruggiero, Elisabetta Teruzzi, Simona Bastonero, Annasilvia Pertusio, Marcello Della Corte, Andrea Sciarrone, Simona Gerocarni Nappo

**Affiliations:** ^1^ Pediatric Urology Unit, Department of Public Health and Pediatric Sciences, Regina Margherita Children’s Hospital, Turin, Italy; ^2^ Division of Urology, Department of Oncology, School of Medicine, San Luigi Gonzaga Hospital, University of Turin, Orbassano, Italy; ^3^ Pediatric Surgery Unit, Department of Women’s and Children’s Health, University of Padua, Padua, Italy; ^4^ Obstetrics-Gynecological Ultrasound and Prenatal Diagnosis Unit, Department of Obstetrics and Gynecology, AOU Città Della Salute e Della Scienza, Turin, Italy

**Keywords:** megacystis, megabladder, oligohydramnios, lower urinary tract obstruction, fetal, prenatal diagnosis, obstructed bladder

## Abstract

**Introduction:**

Diagnosis of prenatal megacystis has a significant impact on the pregnancy, as it can have severe adverse effects on fetal and neonatal survival and renal and pulmonary function. The study aims to investigate the natural history of fetal megacystis, to try to differentiate *in utero* congenital lower urinary tract obstruction (LUTO) from non-obstructive megacystis, and, possibly, to predict postnatal outcome.

**Materials and methods:**

A retrospective single-center observational study was conducted from July 2015 to November 2023. The inclusion criteria were a longitudinal bladder diameter (LBD) >7 mm in the first trimester or an overdistended/thickened-walled bladder failing to empty in the second and third trimesters. Close ultrasound follow-up, multidisciplinary prenatal counseling, and invasive and non-invasive genetic tests were offered. Informed consent for fetal autopsy was obtained in cases of termination of pregnancy or intrauterine fetal demise (IUFD). Following birth, neonates were followed up at the same center. Patients were stratified based on diagnosis: LUTO (G1), urogenital anomalies other than LUTO (“non-LUTO”) (G2), and normal urinary tract (G3).

**Results:**

This study included 27 fetuses, of whom 26 were males. Megacystis was diagnosed during the second and third trimesters in 92% of the fetuses. Of the 27 fetuses, 3 (11.1%) underwent an abortion, and 1 had IUFD. Twenty-three newborns were live births (85%) at a mean gestational age (GA) of 34 ± 2 weeks. Two patients (neonates) died postnatally due to severe associated malformations. Several prenatal parameters were evaluated to differentiate patients with LUTO from those with non-LUTO, including the severity of upper tract dilatation, keyhole sign, oligohydramnios, LBD, and GA at diagnosis. However, none proved predictive of the postnatal diagnosis. Similarly, none of the prenatal parameters evaluated were predictive of postnatal renal function.

**Discussion:**

The diagnosis of megacystis in the second and third trimesters was associated with live births in up to 85% of cases, with LUTO identified as the main cause of fetal megacystis. This potentially more favorable outcome, compared to the majority reported in literature, should be taken into account in prenatal counseling. Megacystis is an often misinterpreted antennal sign that may hide a wide range of diagnoses with different prognoses, beyond an increased risk of adverse renal and respiratory outcomes.

## Introduction

Congenital anomalies of the kidney and urinary tract (CAKUT) are the most common malformations detected during pregnancy, accounting for 20% of all congenital anomalies (1). Hydronephrosis is frequently observed, whereas prenatal megacystis, characterized by overdistension of the bladder, is a less common finding with an estimated prevalence rate ranging from 1:330 to 1:1670 in the first trimester (2). This condition predominantly affects male fetuses and can be identified as early as the first trimester. The fetal bladder is one of the organs visible during ultrasound (US) examination, detectable in 50% of normal fetuses at 10 weeks of gestational age (GA) and in 100% at 13 weeks of gestation (3). It appears as an oval pelvic anechoic structure between the two umbilical arteries, with variable volume throughout the examination (4).

The diagnosis of antenatal megacystis has significant implications for pregnancy, with potential adverse effects on fetal and neonatal survival and renal and pulmonary function. Prenatal counseling is challenging due to the variable etiology of fetal megacystis and the lack of robust scientific evidence regarding prognosis and postnatal outcomes. Enlarged bladders may resolve spontaneously or represent CAKUT, which can be either obstructive or non-obstructive. In congenital lower urinary tract obstruction (LUTO), anatomical bladder outlet obstruction increases bladder and upper urinary tract pressures, potentially leading to renal hypodysplasia, oligohydramnios, and an increased risk of perinatal mortality and poor renal function (5). Non-obstructive megacystis includes several conditions diagnosed postnatally, ranging from chromosomal abnormalities to vesicoureteral reflux (VUR), VACTERL association, and the rare megacystis-microcolon-intestinal hypoperistalsis syndrome (MMIHS) (6). However, not all cases of megacystis progress during pregnancy, with some resolving spontaneously (7, 8). As shown by Fontanella et al. (8), longitudinal bladder diameter (LBD) is a good predictor of resolution in megacystis diagnosed before 18 weeks of gestation. In particular, the ideal LBD cutoff associated with resolution is 12 mm, so LBD could be used to inform the likelihood of spontaneous resolution.

Recently, the European Reference Network for Rare Kidney Diseases developed recommendations for the clinical definition, diagnosis, and management of prenatal LUTO due to the lack of diagnostic features to guide management (9). One of the suggestions that emerged from this study was the need for further researches to objectively define bladder enlargement and to differentiate between obstructive and non-obstructive causes of prenatal megacystis by developing a reliable multiparameter severity scoring system. Indeed, the prenatal differential diagnosis of megacystis can be challenging, as up to one-third of suspected cases may have a non-obstructive cause that is not apparent until postnatal follow-up, making it difficult to provide parents with adequate information about postnatal outcomes (10). There is a need for further clinical validation and adoption of standardized assessment across antenatal centers. To contribute to current knowledge, we report our case series investigating the natural history of fetal megacystis, in an attempt to differentiate *in utero* LUTO from non-obstructive megacystis and possibly predict postnatal outcomes.

## Materials and method

A single-center, retrospective, observational study was conducted involving all consecutive patients diagnosed with fetal megacystis from July 2015 to November 2023. Prenatal US was performed at the Prenatal Diagnostic Center, Gynecological Hospital S. Anna, University Hospital “Città della Salute e della Scienza” of Turin, which is one of the largest women's hospitals in Italy (6,414 childbirths in 2022). Routine US examinations, in accordance with the Italian national prenatal screening program (11), were performed around 20 weeks of gestation using 2D and 3D US (Samsung VS80/Philips Affiniti 70) to screen for malformations and to assess fetal growth. Pregnancies at risk underwent a third-level scan during the first trimester. All examinations were performed by experienced sonographers (AS, SB, and AP).

The inclusion criteria were included the presence of fetal megacystis, which is defined as an LBD of > 7 mm measured from the bladder dome to the bladder neck in the midsagittal plane on an US scan between 11 weeks and 13weeks + 6 days of gestation, or an overdistended/thickened-walled bladder failing to empty in the second and third trimesters of pregnancy. Oligohydramnios was defined as a lower pocket measuring <2 cm and anhydramnios as the absence of amniotic fluid (AF). Hydronephrosis was defined based on the anteroposterior diameter of the pelvis and according to the UTD classification (11). The exclusion criteria included lack of consent, incomplete prenatal data, lack of final diagnosis, and lost to postnatal follow-up.

All enrolled patients (mothers) were informed of the possible use of their data for scientific purposes. All clinical data (Table 1) were obtained with permission from the official hospital software, namely, TrackCARE, and were fully anonymized.

**Table 1:** Maternal and fetal data extracted from TrackCARE.

Prenatal data	Postnatal data
Maternal age	Neonatal weight
Fetal gender	Respiratory distress
Termination of pregnancy (TOP)	Urinary diversion
Intrauterine fetal demise (IUFD)	Kidney and bladder US
Fetal procedures and complications	Associated anomalies (cardiothoracic, skeletal, gastrointestinal, genitalia)
Amniotic fluid	Creatinine at nadir (mg/dl)
Amnioinfusion	Voiding cystourethrography (VCUG): • *Vesicoureteral reflux (VUR)* • *Posterior urethral valves (PUV)* • *Other findings*
Prenatal genetic tests (NIPT, villocentesis, karyotyping, array CGH, others)	Further imaging (i.e., spinal US/MRI)
Gestational age (GA) at diagnosis	Chronic kidney disease (CKD) (dialysis and/or kidney transplant)
Ultrasound (US) data: • *Bladder longitudinal diameter* • *Pelvic AP diameter* • *Ureteral dilatation (mono-/bilateral)* • *Hyperechoic renal parenchyma/cysts* • *Keyhole sign*	History of urinary tract infections (UTI)
Gestational age at delivery	Surgical intervention
Type of delivery	Medication intake (including alpha-blockers and/or anticholinergics)
Fetal autopsy	Follow-up time (months)
	Last follow-up data: • *Creatinine mg/dl* • *US* • *VCUG* • *Uroflowmetry* • *Urodynamic study*
	Neonatal autopsy

After the initial detection of megacystis, the patients were referred to a multidisciplinary prenatal counseling team consisting of gynecologists, geneticists, pediatric urologists, nephrologists, psychologists, and obstetricians. Close follow-up ultrasonography was performed at 2–4 week intervals, and thereafter, according to severity and GA. Detailed US findings, such as bladder diameter, kidney appearance (normal, hydronephrosis, abnormal, renal cortical appearance, ureteral dilatation), amount of AF, presence of associated elements (ascites, urinoma, pulmonary hypoplasia), and associated anomalies, were systematically recorded.

Non-invasive genetic testing (NIPT) and invasive genetic testing, including karyotyping by chorionic villous sample (CVS) or amniocentesis and array CGH, were systematically offered to patients according to GA and performed after informed consent. Voluntary termination of pregnancy (TOP) was regulated by Italian legislation (Law 194/78) and considered up to 22 weeks + 6 days of GA. In cases of TOP or intrauterine fetal death, informed consent was obtained for the fetal autopsy to confirm the diagnosis.

The timing of delivery was determined on a case-by-case basis in consultation with the multidisciplinary prenatal team, and the neonates were cared for in the neonatal unit, neonatal intensive care unit (NICU), pediatric nephrology department, and pediatric urology unit as required. All children were born in the same university hospital.

Based on diagnosis (postnatal or autopsy), patients were divided into:

– Group 1: LUTO, including posterior urethral valves (PUV) and other intravesical anatomical obstructions,
– Group 2: CAKUT other than LUTO, including VUR, VACTERL association, spina bifida, or other diagnoses,
– Group 3: normal urinary tract.

Neonatal outcomes were obtained from medical records and pediatric care notes on the same official hospital software TrackCARE (Table 1), after informed consent.

The primary endpoint was to identify the antenatal signs distinguishing LUTO from non-obstructive megacystis. The secondary endpoint was to investigate the predictive factors for postnatal outcomes and renal function.

Statistical analysis was performed as appropriate: the dichotomous variables were expressed using rates and percentages, whereas the continuous variables were expressed as median unless otherwise stated. Comparative analyses were performed using the Mann–Whitney test for the continuous variables and Fisher's exact test for the categorical variables. *p*-values of <0.05 were considered significant. Statistical analyses were conducted using GraphPad Prism software (version 6, San Diego, CA, USA), which was also used to display tables.

## Results

A retrospective observational study was conducted on 27 consecutive fetuses diagnosed with antenatal megacystis. The GA at diagnosis ranged from 11 weeks + 6 days to 36 weeks + 5 days, with a median of 26 weeks. Of the 27 fetuses, 5 (18%) were diagnosed before 18 weeks of GA (“early megacystis”), and 2 (7%) were diagnosed in the first trimester. The mean maternal age at diagnosis was 31 ± 4 years (range, 22–41 years old). Of the 27 pregnancies, 26 were spontaneous singleton pregnancies, while 1 occurred after *in vitro* fertilization (IVF) (oocyte donation) and was a dichorionic twin pregnancy, with the other female fetus in good health. At diagnosis, the mean longitudinal diameter of the bladder was 40 mm (range, 10.5–80 mm), and the keyhole sign was present in 8 out of the 27 cases (30%).

Of the 27 fetuses, 3 (11%) underwent TOP, and 1 (4%) had intrauterine fetal demise (IUFD). The fetal autopsy of the three TOP cases confirmed the diagnosis of PUV in two cases and prune belly syndrome in one case. One case, initially seen at 19 weeks + 6 days of GA with an LBD of 35 mm, showed spontaneous resolution of the overdistended bladder at 32 weeks of GA, with subsequent confirmation of normal upper and lower urinary tract after birth.

In 17 (63%) of the 27 fetuses, the amount of AF was normal throughout the pregnancy, while in 8 fetuses (30%), oligohydramnios was observed at a mean GA of 23 ± 5 weeks (range, 16–29 weeks). Two (7%) of the 27 fetuses showed polyhydramnios, due to associated gastrointestinal malformation. Among the patients with persistent oligohydramnios, vesicoamniotic shunts (VAS) were placed in three of the eight patients, with AF improvement in two patients and shunt malfunction and dislodgement in one patient.

During pregnancy, 24 (88%) of the 27 patients presented with upper urinary tract anomalies, including hydronephrosis or megaureter in 20 patients, renal dysplasia in 10 patients, and renal agenesis in 1 patient. Associated anomalies were detected in 10 patients (37%), as described in Table 2.

**Table 2:** Prenatal characteristics of the fetuses with megacystis.

Prenatal population features		*N* = 27	%
Amniotic fluid	Anhydramnios/oligohydramnios	8/27	30%
	Polyhydramnios	2/27	7%
	Normal amniotic fluid	17/27	63%
Median gestational age at diagnosis		26 weeks (11 weeks + 6 days—36weeks + 5 days)	
Ultrasound	Ureteral dilatation	20/27 (9 bilateral)	74% (45%)
	Hyperechoic renal parenchyma	10/27	37%
	Bladder longitudinal diameter	40 mm (10.5–80 mm)	
	Keyhole sign	8/27	30%
	TOP	3/27	11%
Course of pregnancy	IUFD	1/27	4%
	Live birth	23/27	85%

Genetic testing, both non-invasive (NIPT/integrated genetic testing) and invasive (array CGH and QF-PCR) tests, was performed in 14 (52%) and 10 (37%) of the 27 patients. Invasive genetic testing was performed after chorionic villus sampling (CVS) in five patients and amniocentesis in eight patients. Four patients who were eligible for invasive genetic tests refused. When prenatal genetic tests were not available, karyotyping, NGS panel, and array CGH were performed after birth.

The prenatal characteristics of the study population are summarized in Table 2.

Twenty-three newborns were live births (85%) at a mean GA of 34 ± 2 weeks (range, 31–39 weeks), with 9 spontaneous deliveries and 14 cesarean sections. All but one neonates were males (96%). Genetic testing (invasive prenatal or postnatal karyotyping) were normal in all live births. One patient with LUTO and severe mental retardation recently underwent next-generation sequencing.

One patient born at 32 weeks + 5 days of GA with prenatal urinoma and severe associated malformations (tracheoesophageal fistula and major cardiac malformation) died on the second day of life. The autopsy confirmed LUTO and pulmonary hypoplasia. Another patient with PUV underwent successful endoscopic valve ablation but died at the age of 4 years due to an unrelated disease (cardiac rhabdomyoma requiring cardiac transplantation).

Timing and mode of delivery, GA, birth weight, respiratory distress, and renal function are summarized in Table 3.

**Table 3:** Postnatal characteristics of the fetuses with megacystis.

Postnatal population features		*N* = 23	%
Type of delivery	Vaginal	9/23	39
	Cesarean	14/23	61
Median gestational age at delivery		34.6 ± 2.7 weeks (31 weeks + 6 days − 39 weeks)	
Gender	Male	22/23	96
	Female	1/23	4
Weight at birth		2,554 g ± 496 g	
Associated anomalies	Skeletal	4/23	17
	Gastrointestinal	3/23	13
	Cardiothoracic	4/23	17
	Genitalia	5/23	21
Adaption anomalies	Respiratory distress at birth	11/23	48
	Need for mechanical ventilation	1/11	9
Surgical procedures after birth	Nephrostomy	1/23	4
	Valve/syringocele ablation	11/23	48
	Vescicostomy/ureterocutaneostomy	5/23	22
Follow-up	Postnatal death	2/23	9
	CKD	5/23	21
	Kidney transplantation	2/23	8
	UTI	10/23	43

Of the 23 live births, 22 underwent voiding cystourethrography (VCUG).

**Figure 1:**
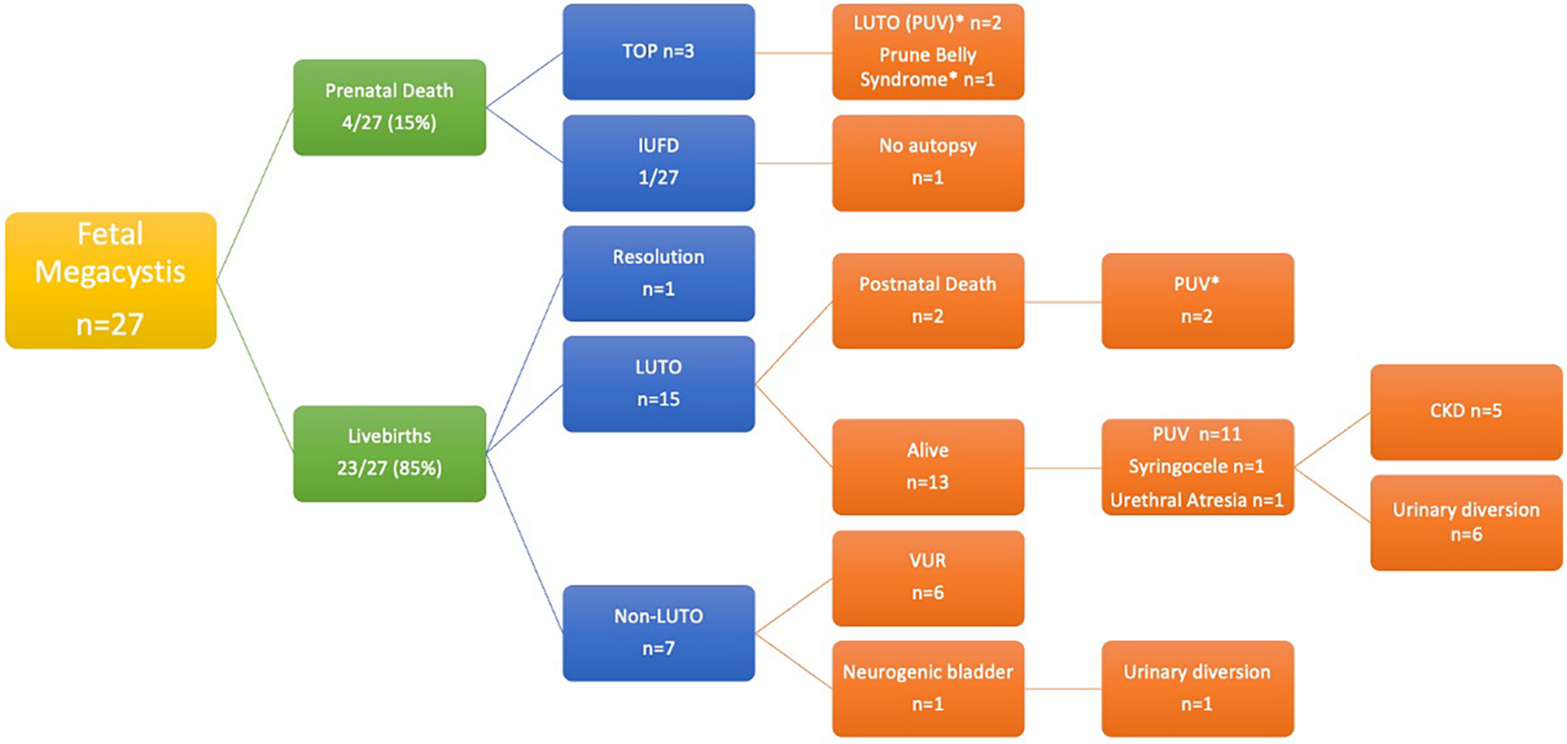
Flow chart of the natural history of the entire cohort. *Diagnosis by autopsy study.

The patients were divided into three groups according to the diagnosis: LUTO (G1), CAKUT other than LUTO (referred to as “non-LUTO”—G2), and normal urinary tract (G3) (Figure 1).

Among the non-LUTO patients, the only female fetus was found to have VUR, and one male fetus with a high-grade VUR in a single kidney was diagnosed with high anorectal malformation and neurogenic bladder.

**Figure 2:**
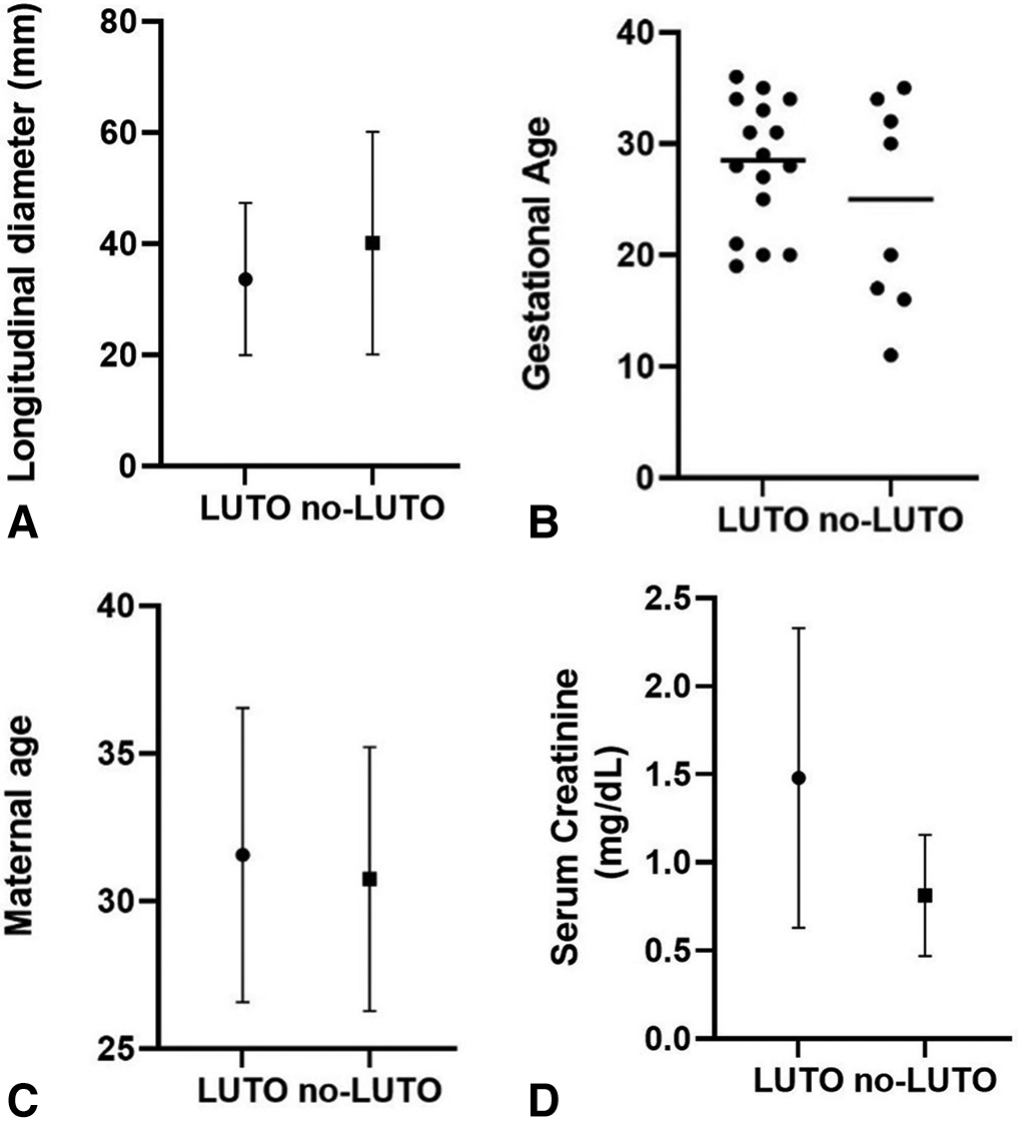
Distribution of longitudinal bladder diameter (**A**), gestational age (**B**), and maternal age (**C**) at diagnosis of megacystis and serum creatinine at nadir (**D**) between the G1 (LUTO) and G2 (no-LUTO) groups.

Several prenatal parameters were evaluated to differentiate patients with LUTO from those with non-LUTO, including the severity of upper tract dilatation, keyhole sign, oligohydramnios, and LBD. However, none of these parameters proved to be predictive of the postnatal diagnosis (Table 4 and Figure 2A). Similarly, GA at diagnosis and maternal age (Figures 2B,C) did not show statistically significant differences between LUTO and non-LUTO (*p = *0.46 and *p = *0.50, respectively).

**Table 4:** US differences between G1 and G2 and their follow-up.

Prenatal features	G1	G2	*p*-value
Ureteral dilatation	11	7	0.36
Keyhole sign	4	2	>0.99
Oligohydramnios	7	1	0.2
Postnatal features	G1	G2	*p*-value
Poor neonatal adaptation (APGAR score of <7 at 5′)	10	1	0.06
Upper urinary tract anomalies	10	6	>0.99
UTI	6	4	0.66
Urinary diversion	6	1	0.19
Chronic kidney disease (CKD)	5	0	0.12

**Figure 3:**
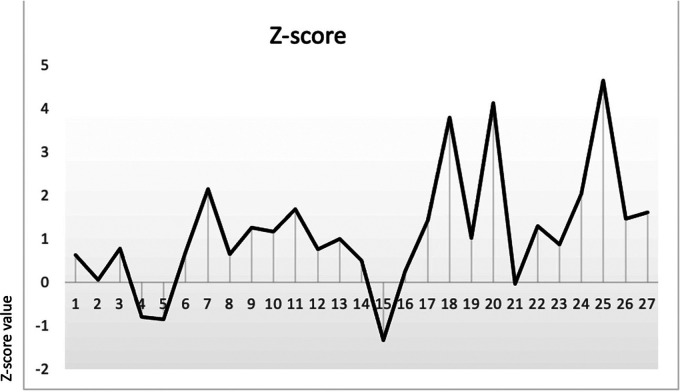
LBD *Z*-score by Fontanella et al. (12) in our sample (SD ± 2.7).

Fontanella et al. (12) reported that LBD Z-scores showed good sensitivity and specificity in differentiating fetuses with PUV (sensitivity, 74%; specificity, 86%). According to their study, we calculated the Z-score (distribution of LBD Z-score in our sample is shown in Figure 3; SD ± 2.7) using the following formula:

$$\;Z\text{-}\mathrm{score}:\;\frac{\mathrm{LBD}\;\;\mathrm{observed}-\mathrm{LBD}\;\;\mathrm{predicted}\;}{\mathrm{SD}}$$

PredictedLBD=(1.48×GA)–17.15=–mm

Mann–Whitney analysis was performed to assess the difference in Z-scores between LUTO and non-LUTO, but no statistical difference was found (*p*-value = 0.3).

**Figure 4:**
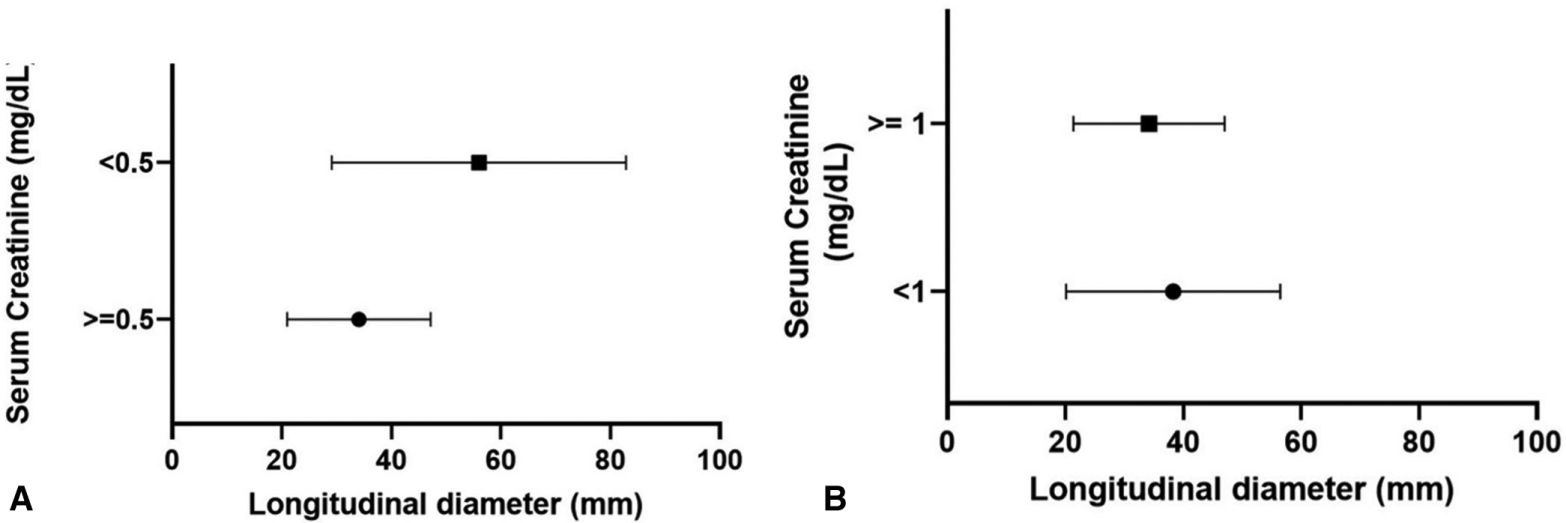
Distribution of longitudinal bladder diameter according to serum creatinine at nadir: 0.5 mg/dl (**A**) and 1 mg/dl (**B**).

Regarding renal function, mean serum creatinine at nadir was 1.4 mg/dl (range, 0.4–3.3 mg/dl), with no significant difference between LUTO and non-LUTO (Figure 2D). None of the prenatal parameters evaluated, such as parenchymal echogenicity, oligohydramnios, keyhole sign, ureteral dilatation, and LBD, had any correlation with the postnatal serum creatinine at nadir (cutoff serum creatinine, 0.5 and 1 mg/dl) (Tables 4, 5 and Figures 4A,B).

**Table 5:** US differences according to serum creatinine.

Prenatal features	Serum creatinine < 1 mg/dl	Serum creatinine ≥ 1 mg/dl	*p*-value
Hyperechoic renal parenchyma	4	5	>0.99
Keyhole sign	3	2	0.62
Low amniotic Fluid	1	7	0.07
Ureteral dilatation	8	8	0.40

At a mean follow-up of 34 months (range, 1–85 months), 5 (24%) of the 23 patients developed chronic kidney disease (CKD): 2 patients underwent renal transplantation, and 2 patients with significant polyuria required overnight bladder drainage. Serum creatinine at 1 year was also compared between LUTO and non-LUTO with no statistical significance difference found. The clinical outcome of LUTO vs. non-LUTO was also compared at the last follow-up and no statistically significant difference was found (Table 4).

## Discussion

Fetal megacystis is a rare anomaly with an estimated first-trimester prevalence ranging from 1:330 to 1:1670 (male-to-female ratio of 8:1) (2). In the first trimester, it is typically defined by a distended/thickened-walled bladder with a longitudinal diameter (LBD)of ≥7 mm or by a median bladder diameter/crown-rump length (CRL) ratio of ≥10% (2). After 14 weeks of gestation, various definitions have been used, the most common being failure of the fetal bladder to empty during a 45 min US scan, although no established cutoff exists (13).

Megacystis is not a diagnosis *per se* but rather a sign that may indicate a range of underlying aetiologies, from LUTO to complex non-obstructive congenital malformations, or it may be a transient phenomenon due to neurological immaturity of the bladder (8). Various authors have investigated different parameters in an attempt to predict the outcome in terms of spontaneous resolution or fetal survival.

Prognosis appears to be largely dependent on factors such as pulmonary development, renal function, GA at diagnosis, underlying etiology, presence of chromosomal abnormalities, and associated malformations.

Chromosomal abnormalities were found by Chen et al. (14) in 10% of 1,088 fetuses with megacystis, the most common being trisomy 13, 18, and 21. Other authors have reported higher figures, with differences depending on the GA at diagnosis or LBD (15). Thus, it is important to offer invasive genetic testing and karyotype analysis, which are essential for correct prenatal counseling and to define fetal prognosis. None of our live births had chromosomal abnormalities, but the limited number of our series may have introduced a bias.

Associated malformations are reported in 24%–40% of cases, with anorectal malformations, VACTERL association, caudal regression syndrome, prune belly syndrome, overgrowth syndrome, and MMIHS being the most common (13). In a large retrospective study of 284 cases, Malin et al. (10) found that 22.2% of cases had associated malformations and that only 56% were alive at 1 year. We observed associated malformations in 37% of megacystis cases, in agreement with the literature. In our series, associated malformations and pulmonary hypoplasia were the main causes of postnatal death.

Several studies have investigated the resolution rate of prenatal megacystis, mostly in patients diagnosed in the first trimester, or in the “early diagnosis” before 18 weeks of GA (8). The study of Iuculano et al. (16) involving 23 fetuses with megacystis and normal karyotype detected in the first trimester reported a favorable outcome in 58% of those with an LBD <15 mm, while the outcome was unfavorable in 100% of those with an LBD >15 mm. Similarly, Kao et al. (17) reported 98 patients diagnosed with megacystsis at 11–14 weeks, 95% of whom had spontaneous resolution at birth: LBD was the best individual predictor of adverse perinatal outcomes, with an LBD ≥12 mm being the optimal cut-point. Lesieur et al. (18) reported that out of 75 megacystis detected in the first trimester, an LBD <12.5 mm was a strong predictor of a favorable outcome, with or without urological problems, with high sensitivity (83.3%) and specificity (87.3%). In a recent metanalysis, Chen et al. (19) reported that almost 40% of cases of megacystis diagnosed at <18 weeks of GA resolved spontaneously *in utero*, compared to 12% of cases diagnosed at >18 weeks of GA. Furthermore, Fontanella et al. (8) found that the prognosis without urological sequelae was good if the resolution of megacystis detected in the first trimester occurred at <23 weeks of GA, whereas later diagnosis and resolution after 23 weeks of GA were associated with a less favorable outcome. We could not confirm these data, as in our series, only 2 of the 27 patients had a diagnosis during the first trimester and none had an LBD <15 mm. The only case of spontaneous resolution in our cohort was diagnosed at 19 weeks of GA, with an LBD of 35 mm, keyhole sign, and thickened bladder wall, with complete resolution at 32 weeks of GA; the urinary tract appeared normal after birth. Overall, the literature suggests that fetuses with megacystis diagnosed in the first trimester (<23 weeks of GA) may have spontaneous resolution with an LBD ranging from <12.5 to 15 mm.

However, outside of this selected population, fetuses with megacystis are generally considered to have a poor outcome. The search for predictive factors for fetal survival and “favorable” or “unfavorable” outcome has yielded inconclusive results. Pulmonary hypoplasia and prematurity are the main causes of mortality in LUTO and are reported in 45% of PUV cases, which may be underestimated due to the “hidden mortality” of undiagnosed fetuses.

As AF after 16 weeks of GA is mostly derived from fetal urine and an adequate AF volume is essential for lung development, fetuses with LUTO and oligohydramnios represent the most severe end of the spectrum, with a high risk of renal dysplasia and pulmonary hypoplasia.

In their meta-analysis, Chen et al. (19) found a significant difference in survival between fetuses with oligohydramnios and those with normal AF (OR of survival with oligohydramnios was 0.24, 95% CI: 0.15–0.37).

Concerns about perinatal survival, respiratory function at birth, and poor future renal function now lead to a high rate of TOP in fetuses with antenatal megacystis. In China, most parents choose to terminate their pregnancy after clinical counseling (19). In France, Lesieur et al. (18) reported that out of 75 megacystis cases detected in the first trimester, 47 (63%) opted for TOP; adding all perinatal deaths to this data, the final live births were only 12 out of 75 (16%). Similarly, Bornes et al. (20) reported 88% of TOP in their series. This was confirmed by Fievet et al. (4), who reported a rate of 66% in TOP. IUFD, TOP, and perinatal death rates reported in recent literature are shown in Table 6.

**Table 6:** Review of published literature in the last decades on the topic of megabladder.

Authors	Country	Study design	Year	Prenatal cases (*n*°)	GA at diagnosis (weeks)	FU (months)	LBD	LUTO	Non-LUTO	Spontaneous resolution	TOP (%)	IUFD (%)	Neonatal death (%)
*Our study*	*Italy*	*Retro*	*2024*	*27*	*26 (11–36 + 5)*	*34 ± 25*	*>7 mm*	*17*	*8*	*1*	*3* (*11)*	*1* (*4)*	*2* (*8)*
Ormonde	Portugal	Retro	2022	43	12.9* ± *0.8	20.6* ± *21.8	≥7 mm	10	17	11	17 (40)	8 (19)	1 (6)
Lesieur	France	Retro	2021	75	12.5* ± *1.79	NS	≥7 mm	31	4	12	47 (63)	11 (15)	5 (7)
Kao	Canada	Retro	2021	98	11–14	1–96	≥7 mm	11	NS	52	38 (39)	7 (7)	NS
Fontanella	Netherlands	Retro	2019	541	11–36	NS	≥7 mm	222	60	23	188 (35)	50 (9)	68 (13)
Iuculano	Italy	Retro	2018	23	11–13	NS	≥7 mm	NS	NS	7	12 (52)	3 (13)	1 (4)
Taghavi	New Zeland	Retro	2017	16	11–32	63.6	≥7 mm	4	2	3	5 (31)	2 (13)	3 (19)
Pellegrino	Italy	Retro	2017	25	12–34	29	>6 mm	17	6	2	1 (4)	0	3 (12)
Tschannen	Switzerland	Retro	2017	53	13–35	24–144	>20 mm in the first half of pregnancy	NS	NS	3	23 (43)	3 (6)	12 (23)
Girard	France	Retro	2016	5	11–12	NS	≥7 mm	NS	NS	5	–	–	–
Fievet	France	Retro	2014	69	11–35	24	>6 mm	27	37	5	23 (33)	5 (7)	NS
Muller	Switzerland	Retro	2014	54	10–39	187.2	≥7 mm	18	3	7	16 (30)	2 (4)	4 (7)
Bornes	France	Retro	2013	84	11–35	12	>7 mm	38	41	5	44 (52)	4 (5)	3 (4)
Al-Hazmi	France	Retro	2012	561	12–36	24	>95th percentile defined for GA	198	NS	NS	307 (55)	15 (3)	7 (1)
Lee	Australia	Retro	2011	61	11–39	NS	>7 mm	39	19	0	30 (49)	17 (28)	NS
Kagan	UK	Retro	2010	35	11–13	NS	≥7 mm	NS	NS	18	6 (17)	1 (3)	NS
Liao	UK	Retro	2003	145	10–14	NS	≥8 mm	8	NS	71	61 (42)	15 (10)	NS

GA, gestational age; FU, follow-up; LBD, longitudinal bladder diameter; LUTO, lower urinary tract obstruction; Non-LUTO, non-lower urinary tract obstruction; TOP, termination of pregnancy; IUFD, intrauterine fetal demise.

Unlike the vast majority of authors, in our series, prenatal death occurred only in four patients (14.8%) with one IUFD at 13 weeks of GA, and three patients undergoing TOP (all observed at < 22 weeks of GA with severe megacystis—LBD of 36, 70, and 80 mm, respectively). An autopsy was performed on three infants, confirming the diagnosis of LUTO in two infants with pulmonary hypoplasia and prune belly syndrome in one infant. Overall, we had a high live birth rate (85%) compared to the literature. Such a good outcome could be explained by the fact that >90% of cases (25/27) were diagnosed in the second and third trimester of pregnancy without chromosomal abnormalities. Our population is similar to the cohort described by Pellegrino et al. (21) who reported only three cases of perinatal death out of 25 cases (12%).

Postnatal follow-up of our patients, which was available in all cases, helped accurately describe the natural history of antenatal megacystis. In accordance with the literature, 63% had LUTO, and 86% of LUTO were PUV (54% of the total cohort), while 32% had a diagnosis other than LUTO, represented by primary VUR (*n* = 6) and VACTERL association (*n* = 1). According to the ESPU-EAU guidelines (22), patients with PUV underwent early endoscopic valve ablation. Urinary diversion was limited to patients with complex associated malformations, very low birth weight, sepsis, or CKD. Primary VUR were all treated conservatively with antibiotic prophylaxis, and treatment (diversion, endoscopy, or surgery) was reserved for patients with recurrent febrile UTI despite prophylaxis or urodynamically high-risk bladders.

As it would be extremely important to identify factors *in utero* that could differentiate LUTO from non-LUTO, several maternal and prenatal US parameters were evaluated, including GA at diagnosis, LBD, keyhole sign (traditionally considered a sign of PUV), hydronephrosis, or ureteral dilatation and oligohydramnios. None of the parameters evaluated proved as a prognostic factor for differentiating LUTO from non-LUTO urinary tract malformations. Fontanella et al. described the *Z*-score as a score that can identify LUTO; unfortunately, we were not able to validate the *Z*-score.

We also investigated possible prognostic factors of postnatal renal function in our cohort; at a median follow-up of 34 months, five patients had CKD. Although not statistically significant, we observed a tendency for patients with LUTO to have adaptative respiratory distress (*p*-value = 0.06), to require prolonged oxygen therapy, and to have elevated serum creatinine at nadir with *p*-value bordering on statistical significance (*p* = 0.05). Fetuses with oligohydramnios tended to have higher serum creatinine at nadir (*p*-value = 0.07). Early in life, elevated serum creatinine levels do not seem to correlate with a higher incidence of chronic renal failure at follow-up (*p*-value = 0.12), but follow-up is short.

The limitations of our study include its retrospective nature and the limited size of the cohort. The strengths of the study included the following: all patients were followed in the same center in a limited time (5 years), with homogeneous and multidisciplinary management, no patient was lost to follow-up, and a final anatomical diagnosis was possible in all patients, even in those undergoing TOP. The natural history of our cohort confirmed that LUTO was the final diagnosis in 63% of megacystis, but contrary to what is generally reported, we observed a high birth rate (85%) and a limited postnatal mortality (8.7%). Overall, our data suggest a more favorable outcome of megacystis diagnosed in the second and third trimesters.

Although CKD was observed in 23.8% of cases at a mean follow-up of 34 months, only 2 patients had end-stage renal disease, and 12 (57%) of the 21 patients older than 3 years were urinary continent. Unfortunately, similar to other authors before us, we could identify neither any predictive factors of postnatal diagnosis of LUTO vs. non-LUTO in our series nor any predictive factors for renal function, but the small number of our cohort was probably the main limiting factor.

In conclusion, megacystis is a prenatal misunderstood sign that may hide a wide spectrum of diagnoses with different prognoses and not only an increased risk of adverse outcomes for renal and respiratory function. Prenatal diagnostic accuracy is essential to identify predictive factors of postnatal outcomes, and a well-structured multidisciplinary approach is fundamental to have a strong impact on pregnancy management and outcomes. This is complex and evolving, influenced by continuous technical improvements in prenatal diagnostics, but for this reason, prospective multicenter studies are required to make sense of the data obtained and to allow for standardization of prenatal counseling and appropriate postnatal management.

## Data Availability

The original contributions presented in the study are included in the article/Supplementary Material; further inquiries can be directed to the corresponding author.
